# Comparison of Costoclavicular Block and Infraclavicular Block Effects on Tissue Oxygen Saturation in Upper Extremity Surgery: A Randomized, Assessor-Blinded Controlled Trial

**DOI:** 10.3390/diagnostics16111715

**Published:** 2026-06-02

**Authors:** Veysi Yazar, Mehmet Baki Bilsel, Tuğba Bingöl Tanriverdi, Ramazan Aslanparçasi, Abdullah Şengül, Mahmut Alp Karahan

**Affiliations:** Department of Anesthesiology and Reanimation, Mehmet Akif İnan Training and Research Hospital, Health Sciences University, Sanliurfa 63040, Türkiye; mehmetbakibilsel@hotmail.com (M.B.B.); tuggbabingol@gmail.com (T.B.T.); rmzn.aslan.2015@gmail.com (R.A.); abdullahsengul342@gmail.com (A.Ş.); mahmutalp_k@yahoo.com (M.A.K.)

**Keywords:** costoclavicular block, infraclavicular block, near-infrared spectroscopy, tissue oxygen saturation, peripheral nerve block

## Abstract

**Background/Objectives**: Ultrasound-guided brachial plexus blocks are widely used in upper extremity surgery. The costoclavicular block (CCB) has been defined in recent years as an alternative to the infraclavicular block (ICB) and attracts attention due to its anatomical advantages. However, studies comparing these two techniques using objective physiological parameters are limited. This study aimed to compare the effectiveness of CCB and ICB techniques by measuring tissue oxygenation with near-infrared spectroscopy (NIRS) and evaluating the role of NIRS in demonstrating block success. **Methods**: Eighty patients undergoing upper extremity surgery were included in this prospective, randomized, comparative study, and the patients were randomly divided into two groups: CCB (*n* = 40) and ICB (*n* = 40). Block success was evaluated using tissue oxygen saturation (StO_2_) and its change (ΔStO_2_) measured by NIRS in addition to sensory and motor assessments. In addition, hemodynamic parameters were recorded. **Results**: A statistically significant increase in StO_2_ and ΔStO_2_ values was observed after block application in both groups. In the costoclavicular block group, the median StO_2_ increased from 78.5 [8.5] at baseline to 86.5 [9.75] at 20 min (*p* < 0.001), while in the infraclavicular block group, it increased from 75.0 [12.0] to 85.0 [9.0] (*p* < 0.001). Similarly, the ΔStO_2_ values increased from 0.0 [0.0] to 9.5 [8.25] in the costoclavicular group and from 0.0 [0.0] to 11.0 [9.0] in the infraclavicular group (both *p* < 0.001). This increase began in the early period and paralleled sensory-motor block findings, indicating that NIRS measurements objectively reflect block success. An isolated intergroup difference in StO_2_ was observed at t10 (83.92 ± 6.17 vs. 80.33 ± 7.31, *p* = 0.023), but no consistent intergroup superiority was demonstrated across the follow-up period. No statistically significant intergroup differences were found for ΔStO_2_ at any time point (*p* > 0.05). The block success rates were similar between groups. The hemodynamic parameters remained stable in both groups, and no clinically significant adverse events were observed. **Conclusions**: CCB and ICB techniques have similar efficacy and safety profiles in upper extremity surgery. NIRS-derived StO_2_ and ΔStO_2_ changes appear to be promising complementary physiological markers associated with block onset. However, the present study was not designed to establish diagnostic cut-off values or predictive accuracy; therefore, NIRS should be interpreted as an adjunct to, rather than a replacement for, conventional sensory and motor block assessment.

## 1. Introduction

In upper extremity surgeries, ultrasound-guided brachial plexus blocks are increasingly preferred over general anesthesia due to their ability to provide effective analgesia and improve perioperative outcomes [1,2].

While the infraclavicular block (ICB) has long been accepted as a safe and effective technique in distal upper extremity surgeries, the costoclavicular block (CCB), described in recent years, offers the potential for faster and more homogeneous block development due to the more compact and superficial arrangement of the brachial plexus cords in this region. However, data comparing the clinical success of these two approaches using objective physiological parameters are limited [3,4,5,6].

In clinical practice, the success of peripheral nerve blocks is generally evaluated through sensory and motor examinations. However, these evaluation methods may be insufficient for the early and objective determination of block efficacy due to their dependence on patient cooperation and their inherently subjective nature [7]. This situation increases the need for methods capable of evaluating block success earlier and quantitatively in regional anesthesia applications.

Near-infrared spectroscopy (NIRS) is an objective monitoring method that enables the non-invasive measurement of tissue oxygenation and can detect microcirculatory changes associated with sympathetic blockade following regional anesthesia at an early stage. Vasodilation and increased regional blood flow following brachial plexus blocks result in a significant increase in tissue oxygen saturation (StO_2_) [8]. Therefore, StO_2_ values measured using the NIRS method can serve as an early and reliable physiological marker for evaluating the efficacy of peripheral nerve blocks. In recent studies, it has been demonstrated that StO_2_ levels in the treated extremity increase significantly starting from the first minutes following a peripheral nerve block [9,10].

The primary objective of this randomized, assessor-blinded comparative trial was to evaluate NIRS-derived tissue oxygenation changes after ultrasound-guided costoclavicular and infraclavicular brachial plexus blocks in patients undergoing upper extremity surgery. The main NIRS-derived outcomes were StO_2_ and ΔStO_2_ over the predefined post-block follow-up period. The secondary outcomes included conventional sensory and motor block assessment at 20 min and peri-procedural hemodynamic stability. The study was designed to determine whether NIRS-derived tissue oxygenation changes could provide complementary physiological information associated with block onset, rather than to establish diagnostic cut-off values for block success.

## 2. Materials and Methods

### 2.1. Ethics

Ethical approval for this study (No. HRÜ/24.04.41) was granted by the Harran University Faculty of Medicine Ethics Committee on 15 April 2024. The study was conducted between April 2024 and April 2025.

### 2.2. Patient Selection

Eighty patients aged 18–65 years with an ASA classification of 1–2 who were scheduled for forearm surgery were included in the study. The selected patients were informed verbally and in writing. Informed consent was obtained from patients who agreed to participate.

Patients who did not wish to participate in the study, patients classified as ASA 3-4-5, patients under 18 or over 65 years of age, patients with coagulation disorders, pregnant or breastfeeding patients, patients using medications that affect tissue oxygen saturation, and patients with neurological sequelae in the arm to be blocked were excluded from the study.

### 2.3. Pre-Block Preparation

On the day of surgery, patients were randomized into two groups by a researcher not involved in the study using a computer-generated random number table. The randomization results were stored in opaque, sealed, and sequentially numbered envelopes. The randomization envelope for each patient was opened immediately before the block was administered. Eighty patients included in the study were divided into two groups: the infraclavicular block (ICB) group and the costoclavicular block (CCB) group.

### 2.4. Costoclavicular Approach

Patients brought to the operating table were monitored using standard non-invasive arterial blood pressure, peripheral oxygen saturation (SpO_2_), and electrocardiography. Tissue oxygen saturation (StO_2_) was measured using a near-infrared spectroscopy device (Root^®^, Masimo Corporation, Irvine, CA, USA). A NIRS probe was placed on the anterior distal aspect of the wrist on the side to be treated in all patients to ensure standardization of measurements. The NIRS probe was connected to a Masimo IC Model RDS7A monitoring device was manufactured by Masimo Corporation (Irvine, CA, USA) to obtain StO_2_ and ΔStO_2_ data. Baseline StO_2_, ΔStO_2_, and hemodynamic data were recorded prior to the procedure. All blocks were administered by experienced anesthesiologists. After baseline values were recorded, the procedure phase began.

To perform the costoclavicular block, the patient was placed in the supine position, and the head was turned to the opposite side; the upper extremity where the block was to be administered was abducted to 90°. Antiseptic (0.5% chlorhexidine) was applied, and the high-frequency linear ultrasound probe (8–12 MHz) of the ultrasound device (Esaote MyLab Seven (Esaote S.p.A., Genoa, Italy)) was placed parallel to the clavicle, just below the latter’s midpoint. The axillary artery and the components of the brachial plexus adjacent to it (lateral, posterior, and medial cords) were identified. For the block procedure, a 22-gauge, 50 mm, insulated bevel-tip needle (B. Braun Stimuplex, Melsungen AG, Melsungen, Germany) was used. While keeping the cords visible, the needle was advanced between the posterior and lateral cords using an in-plane technique, passing through the fascia covering the cords. Following negative aspiration (repeated after every 5 mL injection of local anesthetic), 30 mL of a local anesthetic mixture (15 mL of 0.5% bupivacaine + 15 mL of 2% lidocaine) was injected into this region. After the entire mixture was injected, the spread of the local anesthetics into the cords was observed using ultrasound.

### 2.5. Infraclavicular Approach

To perform the infraclavicular block, the patient was placed in the supine position, the patient’s head was turned to the opposite side, and the upper extremity to be blocked was abducted to 90°. Sterilization was performed with 0.5% chlorhexidine. The high-frequency linear ultrasound probe (8–12 MHz) of the ultrasound device (EsaoteMyLabSeven) was placed directly within the coracoid process to provide optimal imaging, directed to visualize a cross-sectional view of the axillary artery in the parasagittal plane, and the medial–lateral posterior cords were made visible. For the block procedure, a 22-gauge, 50 mm, insulated bevel-tip needle (B. Braun Stimuplex, Melsungen AG, Germany) was used. While keeping the nerves visible, the block needle was advanced toward the nerves surrounding the axillary artery using an in-plane technique. Following negative aspiration (repeated after every 5 mL injection of local anesthetic), 30 mL of local anesthetic mixture (15 mL of 0.5% bupivacaine + 15 mL of 2% lidocaine) was injected into the area to envelop all three cords. After the entire mixture was injected, the spread of the local anesthetics to the nerves was observed via ultrasound.

### 2.6. Clinical Evaluation

The anesthesiologists who performed the block and those who conducted the post-procedure follow-up were different individuals. During the preoperative period, StO_2_, ΔStO_2_, HR, SPO_2_, SBP, and DBP were recorded. For subsequent follow-ups, the 0 min mark was defined as the moment the needle was removed from the skin upon completion of the procedure. Following the block, StO_2_, ΔStO_2_, SPO_2_, HR, SBP, and DBP data were recorded at 0, 5, 10, 15, and 20 min, respectively.

Additionally, 20 min after the block administration, the Bromage scale and pinprick test were performed on the extremity where the block was administered, and the effectiveness of the block was recorded. The Bromage scale assessed whether there was movement in the arm or hand, while the pinprick test evaluated the presence or absence of pain sensation in all relevant dermatomes, and these findings were recorded. Subsequently, the consistency between the objective and subjective tests was verified. Block failure was defined as the need for supplemental anesthesia or conversion to general anesthesia during the procedure.

### 2.7. Statistical Analyses

All statistical analyses in this study were performed using IBM SPSS Statistics for Windows, Version 27.0 (IBM Corp., Armonk, NY, USA). The distribution characteristics of continuous variables were assessed using the Shapiro–Wilk test and visual inspections (histograms, Q–Q plots); variables meeting parametric assumptions are summarized as mean ± SD, while those not normally distributed are summarized as median [IQR]. Categorical variables are presented as *n* (%) and, when appropriate for between-group comparisons, the chi-square or Fisher’s exact test was used; for continuous variables, the independent-samples *t*-test or Mann–Whitney U test was used depending on the distribution. The Friedman test was applied to examine changes over time (Intragroup); when the overall test was significant, pairwise comparisons were performed using the Wilcoxon signed-rank test. Effect sizes are reported as Cohen’s d for parametric comparisons, r = |Z|/√N for non-parametric comparisons, Kendall’s coefficient of concordance (W) for Friedman analyses, and Phi/Cramér’s V for categorical variables; 95% confidence intervals are provided where appropriate. All tests were two-tailed, and the significance level was set at α = 0.05.

The G*Power software (version 3.0.10; Heinrich-Heine-Universität Düsseldorf, Düsseldorf, Germany) was used to evaluate the statistical power and the adequacy of the sample size based on the observed findings. A post hoc power analysis was conducted using the primary outcome, tissue oxygen saturation (StO_2_) at the t10 time point, where the most significant difference was observed (Block Technique 1: 83.92 ± 6.17; Block Technique 2: 80.33 ± 7.31; 95% confidence interval, *p* = 0.023; and effect size d = 0.53). With the current sample size of 72 participants, the achieved statistical power (1 − β) to detect this intergroup difference at an α level of 0.05 was calculated as 0.85. Furthermore, regarding the intragroup changes in oxygenation over time, the Friedman analysis revealed very large effect sizes (Kendall’s W = 0.756–0.836; *p* < 0.001). Given these high effect coefficients, the total sample size of 72 was confirmed to provide a statistical power exceeding 99% to validate the kinetic increase in tissue oxygenation for both techniques. We recruited 40 patients per study group. These power levels and effect sizes demonstrate that the study was statistically sufficient and clinically relevant to support the primary hypotheses.

## 3. Results

**Figure 1:**
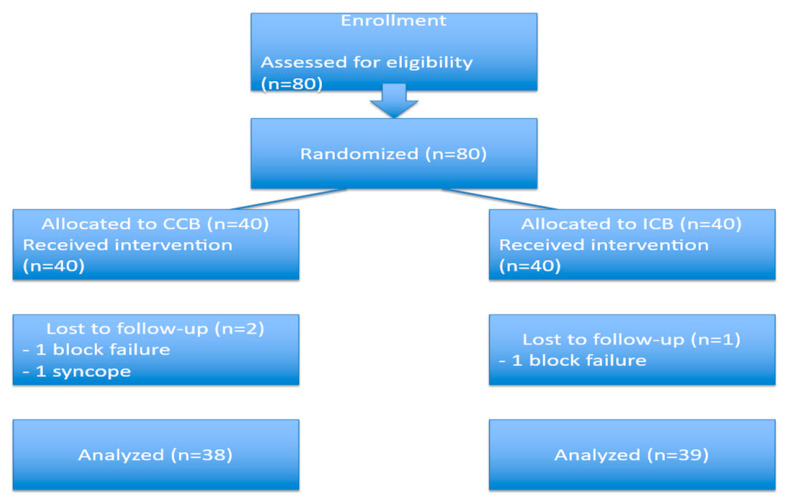
Flow diagram showing patient enrollment, randomization, post-randomization exclusions, and final evaluable analysis population. Of 80 randomized patients, 3 were excluded from the final evaluable analysis: one patient in the infraclavicular block group because of block failure, one patient in the costoclavicular block group because of block failure, and one patient in the costoclavicular block group because of syncope during needle insertion before local anesthetic injection. The final evaluable analysis included 38 patients in the costoclavicular block group and 39 patients in the infraclavicular block group. CCB, costoclavicular block; ICB, infraclavicular block.

Of the 80 randomized patients, 3 were excluded from the final evaluable analysis: one patient in the infraclavicular block group because of block failure, one in the costoclavicular block group because of block failure, and one in the costoclavicular block group because of syncope during needle insertion before local anesthetic injection. Therefore, the final evaluable analysis included 77 patients: 38 in the costoclavicular block group and 39 in the infraclavicular block group (Figure 1).

Baseline demographic, clinical, hemodynamic, and block assessment characteristics are summarized in Table 1. No statistically significant differences were observed between the costoclavicular and infraclavicular block groups in terms of age, sex, body mass index, ASA class, smoking status, diabetes mellitus, hypertension, baseline hemodynamic parameters, baseline StO_2_, Bromage-based motor assessment, or pinprick sensory assessment.

Time-dependent changes in hemodynamic and tissue oxygenation parameters are presented in Table 2. Heart rate, SpO_2_, and MAP showed statistically significant or directional changes at some time points; however, the corresponding effect sizes were very small to low-moderate. In contrast, both StO_2_ and ΔStO_2_ showed marked time-dependent increases after block completion in both groups. The Friedman test demonstrated a significant time effect for StO_2_ and ΔStO_2_ in both block groups, with large effect sizes. In both groups, StO_2_ and ΔStO_2_ initially decreased at t0 compared with baseline and then increased progressively from t5 to t20.

Between-group comparisons of StO_2_ are summarized in Table 3. The mean StO_2_ values tended to be higher in the costoclavicular block group than in the infraclavicular block group at several time points. The most pronounced difference was observed at t10, where StO_2_ was 83.92 ± 6.17% in the costoclavicular block group and 80.33 ± 7.31% in the infraclavicular block group, corresponding to a mean difference of 3.59 percentage points, 95% CI 0.51 to 6.66, *p* = 0.023, and Cohen’s d = 0.530. However, because this was an unadjusted comparison across repeated time points and no consistent intergroup superiority was observed across the entire follow-up period, the t10 finding should be interpreted as exploratory rather than confirmatory evidence of clinical superiority.

Between-group comparisons of ΔStO_2_ are presented in Table 4. No statistically significant intergroup difference was detected in ΔStO_2_ at any time point. The observed effect sizes were very small across the follow-up period, indicating that the magnitude of ΔStO_2_ change was broadly similar between the two block techniques. Therefore, the main signal in ΔStO_2_ was a time-dependent increase after block completion rather than a consistent difference between techniques.

## 4. Discussion

In this study, the effects of ICB and CCB techniques on systemic hemodynamic parameters and tissue oxygenation dynamics (StO_2_ and ΔStO_2_) were compared in patients undergoing upper extremity surgery; additionally, the potential of StO_2_ change to serve as an objective marker for peripheral nerve block success was evaluated. In our study, no significant changes in hemodynamic parameters were observed following the application of block techniques in either patient group; however, a significant increase was detected in NIRS-based StO_2_ and ΔStO_2_ time profiles, though no significant difference was found between the two groups. Similarly, no significant difference was observed in the block success rates between the two groups.

As noted in the literature, near-infrared spectroscopy (NIRS) is an optical monitoring method that enables the non-invasive assessment of oxygenation in biological tissues. In this technique, regional StO_2_ is calculated based on the principle that light in the near-infrared wavelength range is absorbed at different rates by oxyhemoglobin and deoxyhemoglobin [11]. NIRS measurements provide clinically meaningful information in the evaluation of peripheral vascular responses, particularly due to their sensitivity to changes in perfusion and oxygen delivery at the microcirculatory level [12,13]. Vasodilation and increased regional blood flow resulting from the blockade of sympathetic fibers following peripheral nerve blocks lead to an early and marked increase in StO_2_ values measured by NIRS; this makes the NIRS method an objective and early indicator of block success [13,14,15].

**Figure 2:**
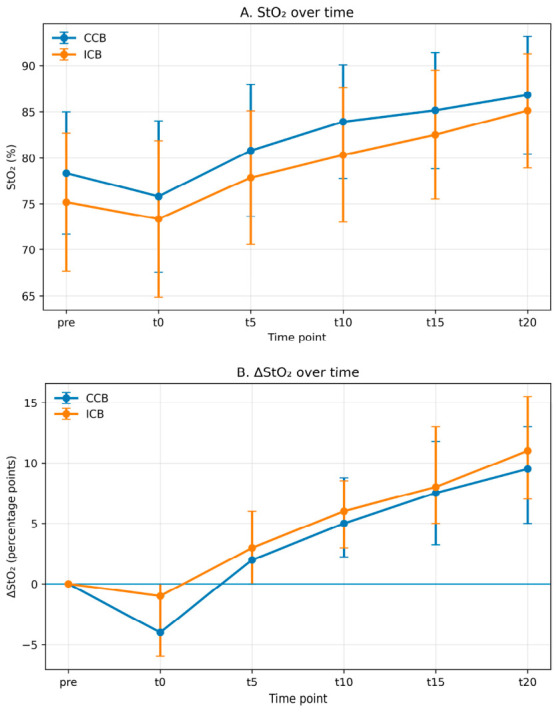
Temporal changes in NIRS-derived tissue oxygen saturation parameters after block completion. Panel (**A**) shows StO_2_ values over time, and Panel (**B**) shows ΔStO_2_ values relative to baseline. Measurements were obtained before block placement and at 0, 5, 10, 15, and 20 min after block completion. CCB, costoclavicular block; ICB, infraclavicular block; NIRS, near-infrared spectroscopy; StO_2_, tissue oxygen saturation; ΔStO_2_, change in tissue oxygen saturation relative to baseline.

As shown in Figure 2, a significant increase in tissue oxygen saturation values was observed following both block techniques, consistent with both physiological mechanisms and previous reports in the literature [9,10]. The observation that StO_2_ values at t0 were lower than baseline may be explained by transient local effects of anesthetic administration, such as tissue compression due to injection volume or an initial vasoconstrictive response, leading to temporary alterations in microcirculation. Data obtained through NIRS-based measurements revealed a marked increase in StO_2_ that began in the early post-block period and persisted up to the 20th minute. However, studies directly comparing StO_2_ changes following different peripheral block techniques are limited in the literature. In our study, however, no significant difference was observed between the two block techniques at any time point. This finding suggests that peripheral nerve block may enhance local microcirculation regardless of the technique used.

One of the key findings of our study is that the increase in StO_2_ may serve as an early and objective indicator for assessing the success of peripheral nerve blocks. It is noteworthy that, in both groups, StO_2_ and ΔStO_2_ values increased consistently with high effect sizes, and this change was parallel to the results of motor and sensory block assessment tests. The fact that block success rates were similar between the groups further supports the notion that the increase in StO_2_ may reflect block success regardless of the technique used.

Although conventional block assessment methods (such as tests based on motor and sensory examination) are easy to administer in clinical practice and are widely used, they are dependent on patient cooperation and can be subjective to a certain extent. Therefore, NIRS-based tissue oxygenation measurements may contribute to clinical practice as an operator-independent, non-invasive, and objective complementary assessment method. However, it is considered that, in daily anesthetic practice, motor and sensory block tests (e.g., pinprick and motor assessment) remain the primary assessment method due to their practicality, whilst NIRS may be used as a supportive tool for assessing block success, particularly in the early stages. It has been reported in the literature that changes in StO_2_ are associated with block success [12,13,14,15], and our study supports these findings.

In our study, although some fluctuations in heart rate, systolic and diastolic arterial pressure, and mean arterial pressure were observed over time in both patient groups following the block procedure, these changes remained limited both statistically and clinically. No clinically significant hypotension, bradycardia, or hemodynamic instability was detected in any patient. The absence of a significant difference between the groups in terms of SpO_2_, mean arterial pressure, and heart rate indicates that both peripheral nerve block techniques are safe in terms of systemic hemodynamic effects.

These findings are consistent with the literature reporting that the effects of peripheral nerve blocks performed in upper extremity surgery on systemic hemodynamics are minimal [16,17]. Similarly, studies evaluating interscalene block also failed to detect permanent or clinically significant hemodynamic changes in the post-block period [18]. In the systematic review and meta-analysis of Lee et al, it has been shown that peripheral nerve blocks contribute to maintaining hemodynamic stability during the perioperative period compared to spinal anesthesia and offer an effective anesthesia approach [19]. This finding can be explained by the fact that, unlike central neuraxial blocks, peripheral nerve blocks do not significantly affect systemic sympathetic tone or venous return.

Despite the limited effect of peripheral nerve blocks on systemic hemodynamics, it is known that they induce significant physiological changes in local circulation in the extremity where they are administered. The strong and sustained increase observed in tissue oxygenation demonstrates that peripheral nerve blocks provide a significant physiological advantage in terms of local circulation and oxygen delivery. In studies using Doppler ultrasound, dilation of arterial vessels and an increase in blood flow velocity have been detected in the relevant extremity following axillary, ICB, and CCB procedures [20,21]. These changes are associated with vasodilation and increased regional perfusion resulting from the blockade of sympathetic fibers. The statistically significant difference observed at t10 (*p* = 0.023) should be interpreted with caution, because it may lose significance after correction for multiple comparisons (e.g., Bonferroni correction).

### Limitations

This study has certain limitations. The inclusion of only hemodynamically stable patients (ASA I–II) undergoing elective upper limb surgery may limit the generalizability of the findings to high-risk populations with impaired vascular function. Crucially, the low rate of block failure in our study population precluded a robust analysis of diagnostic accuracy. Consequently, we were unable to perform ROC curve analysis to establish optimal cut-off values, sensitivity, specificity, or predictive power for StO_2_. Therefore, whilst our results demonstrate significant trends, the definitive clinical threshold for distinguishing successful from failed blocks remains to be validated in larger trials with higher failure rates.

## 5. Conclusions

This study demonstrates that peripheral nerve blocks administered during upper limb surgery can be safely performed without adversely affecting systemic hemodynamics and are associated with an increase in local tissue oxygenation. The changes observed in StO_2_ values obtained via NIRS suggest a potential correlation between tissue oxygenation and the success of the block. The similar increase in tissue oxygenation observed in both block techniques indicates comparable physiological effects in terms of efficacy and safety. However, whilst NIRS-based StO_2_ monitoring appears to be a promising non-invasive parameter, further studies with sufficient power are required to determine its clinical utility in assessing the success of peripheral nerve blocks.

## Figures and Tables

**Table 1:** Baseline demographic, clinical, hemodynamic, and block assessment characteristics according to study group.

Variable	CCB (*n* = 38)	ICB (*n* = 39)	*p*-Value
Age, years	29.5 [22.0]	34.0 [24.0]	0.610
Female sex, *n* (%)	12 (31.6)	8 (20.5)	0.307
BMI, kg/m^2^	27.36 ± 4.76	26.87 ± 4.62	0.649
ASA class I/II, *n* (%)	12/26 (31.6/68.4)	13/26 (33.3/66.7)	0.869
Smoking, *n* (%)	17 (44.7)	22 (56.4)	0.306
Diabetes mellitus, *n* (%)	5 (13.2)	3 (7.7)	0.481
Hypertension, *n* (%)	4 (10.5)	2 (5.1)	0.431
Baseline HR, bpm	77.71 ± 15.11	76.87 ± 15.15	0.808
Baseline SpO_2_, %	98.0 [2.25]	98.0 [2.00]	0.909
Baseline SBP, mmHg	134.0 [22.5]	132.0 [23.0]	0.498
Baseline DBP, mmHg	81.08 ± 11.05	77.62 ± 11.84	0.189
Baseline MAP, mmHg	101.5 [19.5]	96.0 [19.0]	0.064
Baseline StO_2_, %	78.34 ± 6.65	75.18 ± 7.50	0.054
Bromage category, *n* (%)	10 (26.3)/19 (50.0)/9 (23.7)	12 (30.8)/19 (48.7)/8 (20.5)	0.892
Pinprick category, *n* (%)	37 (97.4)/1 (2.6)	35 (89.7)/4 (10.3)	0.358

CCB, costoclavicular block; ICB, infraclavicular block; BMI, body mass index; ASA, American Society of Anesthesiologists; HR, heart rate; SpO_2_, peripheral oxygen saturation; SBP, systolic blood pressure; DBP, diastolic blood pressure; MAP, mean arterial pressure; StO_2_, tissue oxygen saturation.

**Table 2:** Time-dependent changes in hemodynamic and tissue oxygenation parameters after costoclavicular and infraclavicular block.

Parameter	Group	pre	t0	t5	t10	t15	t20	Friedman *p*-Value	Kendall’s W
HR, bpm	CCB	76 [16.50]	79 [14.50]	76.5 [18.25]	76.5 [17.75]	75.5 [18.25]	77.5 [15.25]	0.032	0.064
HR, bpm	ICB	75 [16.00]	77 [18.00]	71 [16.00]	72 [15.00]	73 [15.00]	74 [12.00]	0.003	0.091
SpO_2_, %	CCB	98 [2.25]	98 [3.00]	97 [3.00]	97 [3.00]	97.5 [3.00]	98 [2.25]	0.115	0.047
SpO_2_, %	ICB	98 [2.00]	98 [2.00]	98 [3.00]	98 [3.00]	98 [3.00]	98 [3.00]	0.212	0.036
MAP, mmHg	CCB	101.5 [19.50]	100 [18.00]	98.5 [20.00]	96 [18.00]	98.5 [19.50]	101 [20.50]	0.002	0.100
MAP, mmHg	ICB	96 [19.00]	100 [17.00]	95 [16.00]	91 [12.00]	93 [14.00]	96 [14.00]	<0.001	0.155
StO_2_, %	CCB	78.5 [8.50]	76.0 [12.50]	80.0 [11.75]	84.0 [8.00]	85.0 [8.00]	86.5 [9.75]	<0.001	0.795
StO_2_, %	ICB	75.0 [12.00]	74.0 [15.00]	79.0 [12.00]	81.0 [11.00]	84.0 [7.00]	85.0 [9.00]	<0.001	0.756
ΔStO_2_, percentage points	CCB	0.0 [0.00]	−4.0 [5.25]	2.0 [6.00]	5.0 [7.00]	7.5 [9.00]	9.5 [8.25]	<0.001	0.836
ΔStO_2_, percentage points	ICB	0.0 [0.00]	−1.0 [6.00]	3.0 [6.00]	6.0 [6.00]	8.0 [9.00]	11.0 [9.00]	<0.001	0.756

Data are presented as median [IQR]. Intragroup time-dependent changes were analyzed using the Friedman test, and effect size was reported as Kendall’s coefficient of concordance (W). CCB, costoclavicular block; ICB, infraclavicular block; HR, heart rate; SpO_2_, peripheral oxygen saturation; MAP, mean arterial pressure; StO_2_, tissue oxygen saturation; ΔStO_2_, change in tissue oxygen saturation relative to baseline.

**Table 3:** Intergroup comparison of StO_2_ values over time.

Time	CCB (*n* = 38)	ICB (*n* = 39)	Mean Difference	95% CI	t (df)	*p*-Value	Cohen’s d	95% CI for d
pre	78.34 ± 6.65	75.18 ± 7.50	3.16	−0.06 to 6.38	1.956 (75)	0.054	0.446	−0.008 to 0.897
t0	75.79 ± 8.23	73.36 ± 8.50	2.43	−1.37 to 6.23	1.275 (75)	0.206	0.291	−0.160 to 0.739
t5	80.82 ± 7.17	77.85 ± 7.25	2.97	−0.31 to 6.24	1.806 (75)	0.075	0.412	−0.041 to 0.862
t10	83.92 ± 6.17	80.33 ± 7.31	3.59	0.51 to 6.66	2.324 (75)	0.023	0.530	0.073 to 0.983
t15	85.16 ± 6.32	82.51 ± 7.00	2.65	−0.38 to 5.67	1.739 (75)	0.086	0.396	−0.056 to 0.846
t20	86.82 ± 6.36	85.13 ± 6.20	1.69	−1.17 to 4.54	1.178 (75)	0.242	0.269	−0.181 to 0.717

Data are presented as mean ± SD. Between-group comparisons were performed using the independent-samples *t*-test. Effect size is reported as Cohen’s d with 95% CI. The t10 comparison was statistically significant in the unadjusted analysis; however, because multiple time-point comparisons were performed, this finding should be interpreted as exploratory. CCB, costoclavicular block; ICB, infraclavicular block; StO_2_, tissue oxygen saturation; CI, confidence interval; SD, standard deviation.

**Table 4:** Intergroup comparison of ΔStO_2_ values over time.

Time	CCB (*n* = 38)	ICB (*n* = 39)	U	Z	*p*-Value	Effect Size r
pre	0.0 [0.00]	0.0 [0.00]	741.0	0.000	1.000	0.000
t0	−4.0 [5.25]	−1.0 [6.00]	631.5	−1.120	0.263	0.128
t5	2.0 [6.00]	3.0 [6.00]	726.0	−0.153	0.878	0.017
t10	5.0 [7.00]	6.0 [6.00]	697.0	−0.449	0.653	0.051
t15	7.5 [9.00]	8.0 [9.00]	664.0	−0.786	0.432	0.090
t20	9.5 [8.25]	11.0 [9.00]	612.0	−1.317	0.188	0.150

Data are presented as median [IQR]. Between-group comparisons were performed using the Mann–Whitney U test. Effect size was reported as r. No statistically significant intergroup difference in ΔStO_2_ was observed at any time point. CCB, costoclavicular block; ICB, infraclavicular block; ΔStO_2_, change in tissue oxygen saturation relative to baseline.

## Data Availability

The original contributions presented in this study are included in the article; further inquiries can be directed to the corresponding author.

## Abbreviations

The following abbreviations are used in this manuscript:

CCB	Costoclavicular block
ICB	Infraclavicular block
NIRS	Near-infrared spectroscopy
StO2	Tissue oxygen saturation
ΔStO2	Tissue oxygen saturation change
